# 

**Published:** 1913-11-15

**Authors:** 


### CONTENTS.

| | PAGE |
|---|---|
| The Best Side of a Man ... ... ... ... ... | 155 |
| Staff Appointments at the Cardiff Medical School... ... ... ... ... ... ... | 156 |
| Hospital and Institutional News, including— London Clubs in Time of War—The Lady with the Clear Head—Women Doctors and Mental Hospitals—The Public and Poor-Law Infirmaries—Brentford Guardians' Latest Decision—Medical Policy and Sanatorium Patients—Where are your Brains? | 157 |
| Modern Orthopædy: Recent Advances and What they Mean ... ... ... ... ... ... | 163 |
| The Real Character of the Lady with the Lamp | 165 |
| The British Attitude Towards Vaccination Laws ... ... ... ... ... ... ... ... | 166 |
| Garden Cities for Consumptives ... ... ... By J. E. ESSLEMONT, M.B. | 167 |
| A Lesson in Self-Experimentation ... ... | 170 |
| Reports on Some Cottage Hospitals in Suffolk and Cambs. ... ... ... ... ... ... ... By Sir HENRY BURDETT, K.C.B., K.C.V.O. | 171 |
| Contributions from Approved Societies to Hospitals ... ... ... ... ... ... ... | 173 |
| Dr. Keats on Infirmary Discipline ... ... | 175 |
| The Secrets of Economy in Institutional Housekeeping ... ... ... ... ... | 177 |
| Opening of the Marple Cripples' Home ... | 179 |
| An Interesting Career ... ... ... ... | 179 |
| The Non-Panel Movement ... ... ... ... Free Choice of Herbalist. | 180 |
| The Institutional Worker ... ... (Suppl.) | 1 |
| Our Bureau of Information ... ... ,, | 2 |
| Nursing Homes ... ... ... ... ,, | 3 |
| Buildings Contemplated and in Progress ,, | 3 |
| The Book Market ... ... ... ,, | 3 |

